# Supplementary material for: GRAM domain proteins specialize functionally distinct ER-PM contact sites in human cells
Source: eLife. 2018 Feb 22;7:e31019. doi: 10.7554/eLife.31019 (PMC5823543; doi:10.7554/eLife.31019)
Supplement: Figure 2—source data 1. — Bottom table is corresponding two-tailed t-test values. [file elife-31019-fig2-data1.docx]

**Figure 2 – Source Data 1**

**Figure 2D Bar Graph**

**Co-localization Analysis**

|  | % Pixel Overlap |
| --- | --- |
| GRAMD2a with E-Syt2 | 75.9 ± 2.5 % |
| GRAMD2a with E-Syt3 | 85.7 ± 3.3 % |
| GRAMD1a with E-Syt2 | 8.9 ± 1.8 % |
| GRAMD1a with E-Syt3 | 8.9 ± 1.0 % |

|  | % Pixel Overlap |
| --- | --- |
| E-Syt2 with GRAMD2a | 47.6 ± 5.8 % |
| E-Syt2 with GRAMD1a | 1.4 ± 0.3 % |
| E-Syt3 with GRAMD2a | 52.8 ± 3.6 % |
| E-Syt3 with GRAMD1a | 1.9 ± 0.4 % |

**Two-tailed T-test**

|  | P-value |
| --- | --- |
| GRAMD2a with E-Syt2 vs GRAMD2a with E-Syt3 | 0.823269629 |
| GRAMD1a with E-Syt1 vs GRAMD1a with E-Syt3 | 0.993195528 |
| GRAMD2a with E-Syt2 with GRAMD1a with E-Syt2 | 8.97463E-12 |
| GRAMD2a with E-Syt3 with GRAMD1a with E-Syt3 | 3.40272E-17 |

|  | P-value |
| --- | --- |
| E-Syt2 with GRAMD2a vs E-Syt3 with GRAMD2a | 0.813905889 |
| E-Syt2 with GRAMD1a vs E-Syt3 with GRAMD1a | 0.383612438 |
| E-Syt2 with GRAMD2a vs E-Syt2 with GRAMD1a | 9.56183E-07 |
| E-Syt3 with GRAMD2a vs E-Syt3 with GRAMD1a | 3.14338E-13 |
