# Supplementary material for: GRAM domain proteins specialize functionally distinct ER-PM contact sites in human cells
Source: eLife. 2018 Feb 22;7:e31019. doi: 10.7554/eLife.31019 (PMC5823543; doi:10.7554/eLife.31019)
Supplement: Figure 4—source data 1. [file elife-31019-fig4-data1.docx]

**Figure 4 – Source Data 1**

**Figure 4D Bar Graph**

**Liposome binding assay with recombinant GRAMD2a**

Values below are % of GRAMD2a that pelleted with liposomes as compared to total GRAMD2a using in liposome binding assay.

| **% Lipid in Liposomes** | **PI(4)P** | **PI(4,5)P2** |
| --- | --- | --- |
| 1% | 15.9 ± 3.9 % | 32.7 ± 4.3 % |
| 5% | 34.5 ± 6.9 % | 64.5 ± 6.5 % |
| 15% | 96.1 ± 0.9 % | 94.7 ± 0.7 % |

Liposomes containing 85% PC and 15 % PS: 1.0 ± 0.6 %

Liposomes containing 85% PC and 15 % PI: 1.4 ± 0.8 %
