# Supplementary material for: GRAM domain proteins specialize functionally distinct ER-PM contact sites in human cells
Source: eLife. 2018 Feb 22;7:e31019. doi: 10.7554/eLife.31019 (PMC5823543; doi:10.7554/eLife.31019)
Supplement: Figure 5—source data 1. — 1 μM TG is added at T = 30 s. Standard Error shown. Analysis was performed on n = 12 cells from three independent experiments. [file elife-31019-fig5-data1.docx]

**FIGURE 5 – Source Data 1**

**FIGURE 5 – TABLE 1**

| **Time (s)** | **% D2 with STIM1** | **% STIM1 with D2** |
| --- | --- | --- |
| 0 | 0.58 ± 0.38% | 18.34 ± 1.32% |
| 50 | 5.48 ± 4.66% | 22.42 ± 1.07% |
| 100 | 17.61 ± 3.86% | 45.98 ± 11.27% |
| 120 | 30.63 ± 2.57% | 59.24 ± 10.26% |
| 130 | 45.75 ± 6.02% | 77.09 ± 4.30% |
| 140 | 57.51 ± 2.14% | 68.24 ± 1.36% |
| 150 | 69.33 ± 9.54% | 56.16 ± 5.25% |
| 160 | 69.53 ± 4.64% | 47.61 ± 6.69% |
| 200 | 71.13 ± 6.32% | 39.33 ± 3.73% |
| 250 | 71.17 ± 7.62% | 36.23 ± 6.35% |

**Figure 5 – Table 1 Legend**: Quantification of the percentage of co-localized total fluorescent pixels of GRAMD2a-GFP with mCherry-STIM1 or mCherry-STIM1 with GRAMD2a-GFP as a function of time after TG addition. 1 μM TG is added at T=30s. Standard Error shown. Analysis was performed on n=12 cells from three independent experiments.
