# Supplementary material for: GRAM domain proteins specialize functionally distinct ER-PM contact sites in human cells
Source: eLife. 2018 Feb 22;7:e31019. doi: 10.7554/eLife.31019 (PMC5823543; doi:10.7554/eLife.31019)
Supplement: Figure 5—source data 2. — Quantification of 12 cells shown in Figure 5—source data 1. Figure 5—figure supplement 1D Bar Graph: Top table is pixel overlap of GRAMD2a and GRAMD1a with STIM1; standard Error shown. Bottom table is corresponding two-tailed t-test values. [file elife-31019-fig5-data2.docx]

**Figure 5 – Source Data 2**

**Figure 5B Representative line graph**

Total pixels of GRAMD2a-eGFP and mCherry-STIM1 fluorescence during TG-treatment experiments (1 μM TG is added at T=30s).

| Time | GRAMD2 | STIM1 |
| --- | --- | --- |
| 0 | 5135 | 67 |
| 50 | 5238 | 252 |
| 100 | 5202 | 1283 |
| 120 | 5014 | 1947 |
| 130 | 5081 | 2172 |
| 140 | 4986 | 3766 |
| 150 | 5098 | 4777 |
| 160 | 5089 | 5914 |
| 200 | 4985 | 8033 |
| 250 | 5075 | 8306 |

**Figure 5 – Supplement 1D Bar Graph**

**Co-localization Analysis**

|  | % Pixel Overlap |
| --- | --- |
| GRAMD2a with STIM1 | 80.9 ± 3.0 % |
| GRAMD1a with STIM1 | 7.7 ± 1.2 % |
| STIM1 with GRAMD2a | 34.6 ± 3.2 % |
| STIM1 with GRAMD1a | 4.2 ± 1.0 % |

**Two-tailed T-test**

|  | P-value |
| --- | --- |
| GRAMD2a with STIM1 with GRAMD1a with STIM1 | 4.02337E-13 |
| STIM1 with GRAMD2a vs STIM1 with GRAMD1a | 5.00425E-06 |
