# Supplementary material for: GRAM domain proteins specialize functionally distinct ER-PM contact sites in human cells
Source: eLife. 2018 Feb 22;7:e31019. doi: 10.7554/eLife.31019 (PMC5823543; doi:10.7554/eLife.31019)
Supplement: Figure 6—source data 1. — Bottom table is corresponding two-tailed t-test values. [file elife-31019-fig6-data1.docx]

**Figure 6 – Source Data 1**

**Figure 6B Bar Graph**

**Co-localization Analysis**

|  | % Pixel Overlap |
| --- | --- |
| GRAMD2a with STIM1 | 80.9 ± 3.0 % |
| GRAMD2 with STIM1ΔK | 16.0 ± 3.4 % |
| GRAMD1a with STIM1 | 7.7 ± 1.2 % |
| GRAMD1a with STIM1ΔK | 17.3 ± 3.6 % |

|  | % Pixel Overlap |
| --- | --- |
| STIM1 with GRAMD2 | 34.6 ± 3.2 % |
| STIM1 with GRAMD1a | 4.2 ± 1.0 % |
| STIM1ΔK with GRAMD2 | 10.4 ± 3.9 % |
| STIM1ΔK with GRAMD1a | 15.2 ± 3.0 % |

**Two-tailed T-test**

|  | P-value |
| --- | --- |
| GRAMD2a with STIM1 vs GRAMD2a with STIM1ΔK | 4.4729E-12 |
| GRAMD1a with STIM1 vs GRAMD1a with STIMΔK | 0.027042273 |
| GRAMD2a with STIM1 with GRAMD1a with STIM1 | 4.02337E-13 |
| GRAMD2a with STIM1ΔK with GRAMD1a with STIM1ΔK | 0.789093072 |

|  | P-value |
| --- | --- |
| STIM1 with GRAMD2a vs STIM1ΔK with GRAMD2a | 0.000166216 |
| STIM1 with GRAMD1a vs STIM1ΔK with GRAMD1a | 0.004953563 |
| STIM1 with GRAMD2a vs STIM1 with GRAMD1a | 5.00425E-06 |
| STIM1ΔK with GRAMD2a vs STIM1ΔK with GRAMD1a | 0.364635049 |
